# Robustness of single-cell RNA-seq for identifying differentially expressed genes

**DOI:** 10.1186/s12864-023-09487-y

**Published:** 2023-07-03

**Authors:** Yong Liu, Jing Huang, Rajan Pandey, Pengyuan Liu, Bhavika Therani, Qiongzi Qiu, Sridhar Rao, Aron M. Geurts, Allen W. Cowley, Andrew S. Greene, Mingyu Liang

**Affiliations:** 1 Department of Physiology, Center of Systems Molecular Medicine, Medical College of Wisconsin, Milwaukee, WI USA; 2 Department of Physiology, University of Arizona College of Medicine – Tucson, Tucson, AZ USA; 3 Key Laboratory of Precision Medicine in Diagnosis and Monitoring Research of Zhejiang Province, Sir Run Run Shaw Hospital, Zhejiang University School of Medicine, Hangzhou, China; 4 Cancer Center, Zhejiang University, Hangzhou, China; 5 Institute of Translational Medicine, Zhejiang University School of Medicine, Hangzhou, China; 6 Versiti Blood Research Institute, Milwaukee, WI USA; 7 Department of Cell Biology, Neurobiology, and Anatomy, Medical College of Wisconsin, Milwaukee, WI USA; 8 Division of Pediatric Hematology/Oncology/Transplantation, Medical College of Wisconsin, Milwaukee, WI USA; 9 The Jackson Laboratory, Bar Harbor, ME USA

**Keywords:** RNA-seq, Gene expression, Stem cell, Single cell

## Abstract

**Background:**

A common feature of single-cell RNA-seq (scRNA-seq) data is that the number of cells in a cell cluster may vary widely, ranging from a few dozen to several thousand. It is not clear whether scRNA-seq data from a small number of cells allow robust identification of differentially expressed genes (DEGs) with various characteristics.

**Results:**

We addressed this question by performing scRNA-seq and poly(A)-dependent bulk RNA-seq in comparable aliquots of human induced pluripotent stem cells-derived, purified vascular endothelial and smooth muscle cells. We found that scRNA-seq data needed to have 2,000 or more cells in a cluster to identify the majority of DEGs that would show modest differences in a bulk RNA-seq analysis. On the other hand, clusters with as few as 50–100 cells may be sufficient for identifying the majority of DEGs that would have extremely small *p* values or transcript abundance greater than a few hundred transcripts per million in a bulk RNA-seq analysis.

**Conclusion:**

Findings of the current study provide a quantitative reference for designing studies that aim for identifying DEGs for specific cell clusters using scRNA-seq data and for interpreting results of such studies.

**Supplementary Information:**

The online version contains supplementary material available at 10.1186/s12864-023-09487-y.

## Background

Using single-cell RNA-seq (scRNA-seq) data to identify differentially expressed genes (DEGs) between cell types or for a specific cell type between experimental conditions is potentially a powerful approach as many cell types are difficult or impossible to purify physically. Several analytical methods are available for identifying DEGs using scRNA-seq data [1–7].

A major and common feature of scRNA-seq data is that the number of cells of each cell type may vary widely, ranging from a few dozen to several thousand. The characteristics of DEGs, such as the consistency and magnitude of differential expression and the transcript abundance, also vary widely. It is not clear whether scRNA-seq data from a small number of cells allow robust identification of DEGs with various characteristics. This is a critical question as the number of studies using scRNA-seq data to identify DEGs is exploding.

## Results

**Fig. 1:**
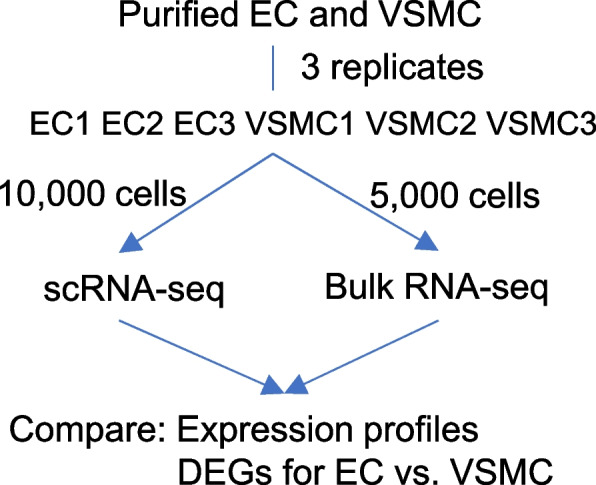
Study outline. The 3 replicates referred to 3 separate rounds of differentiation of an iPSC line. Cells collected from each round of differentiation were split for scRNA-seq and bulk RNA-seq analyses. EC, iPSC-derived endothelial cells; VSMC, iPSC-derived vascular smooth muscle cells; RNA-seq, poly(A)-dependent RNA-seq; DEG, differentially expressed gene

**Fig. 2:**
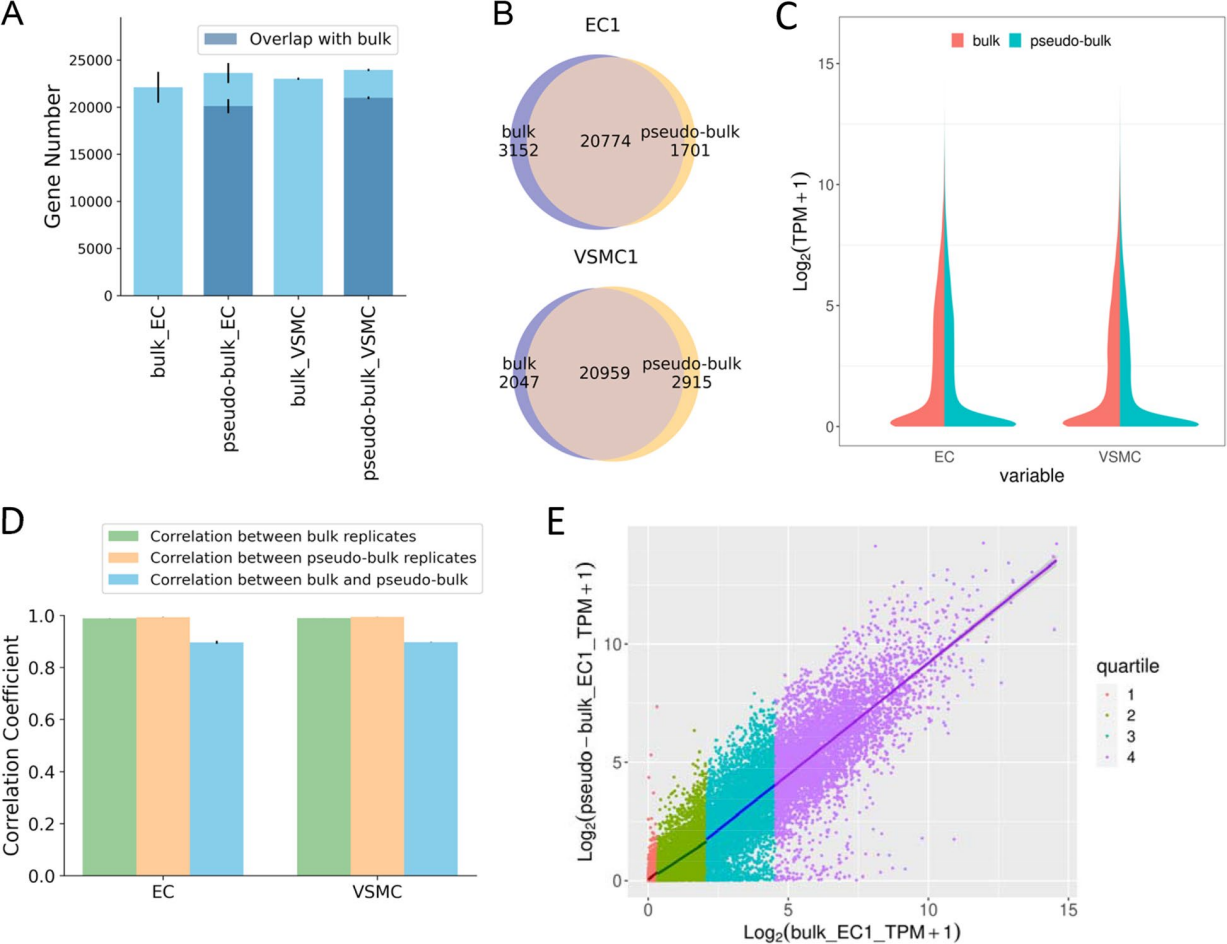
Transcriptome profiles from bulk and pseudo-bulk RNA-seq are modestly correlated. **A** Number of genes detected by bulk and pseudo-bulk, and the overlaps. **B** Venn diagram for examples of panel A. **C** log_2_(TPM + 1) of genes detected in both bulk and pseudo-bulk data. **D** Correlations between bulk and pseudo-bulk data. Abundance of genes not detected in one of the two datasets was set to 0 TPM. **E** An example of the correlation. Genes were divided into quartiles based on the abundance in bulk RNA-seq. EC, iPSC-derived endothelial cells; VSMC, iPSC-derived vascular smooth muscle cells; TPM, transcripts per million

We addressed this question by performing scRNA-seq and poly(A)-dependent bulk RNA-seq in comparable aliquots of human induced pluripotent stem cells (iPSC)-derived, purified vascular endothelial and smooth muscle cells (EC and VSMC) (Fig. 1; Supplemental Figure S1; Supplemental Tables S1 and S2). scRNA-seq data were analyzed directly or aggregated to produce pseudo-bulk RNA-seq data. The overall transcript profile based on pseudo-bulk RNA-seq was modestly consistent with bulk RNA-seq (Fig. 2).

**Fig. 3:**
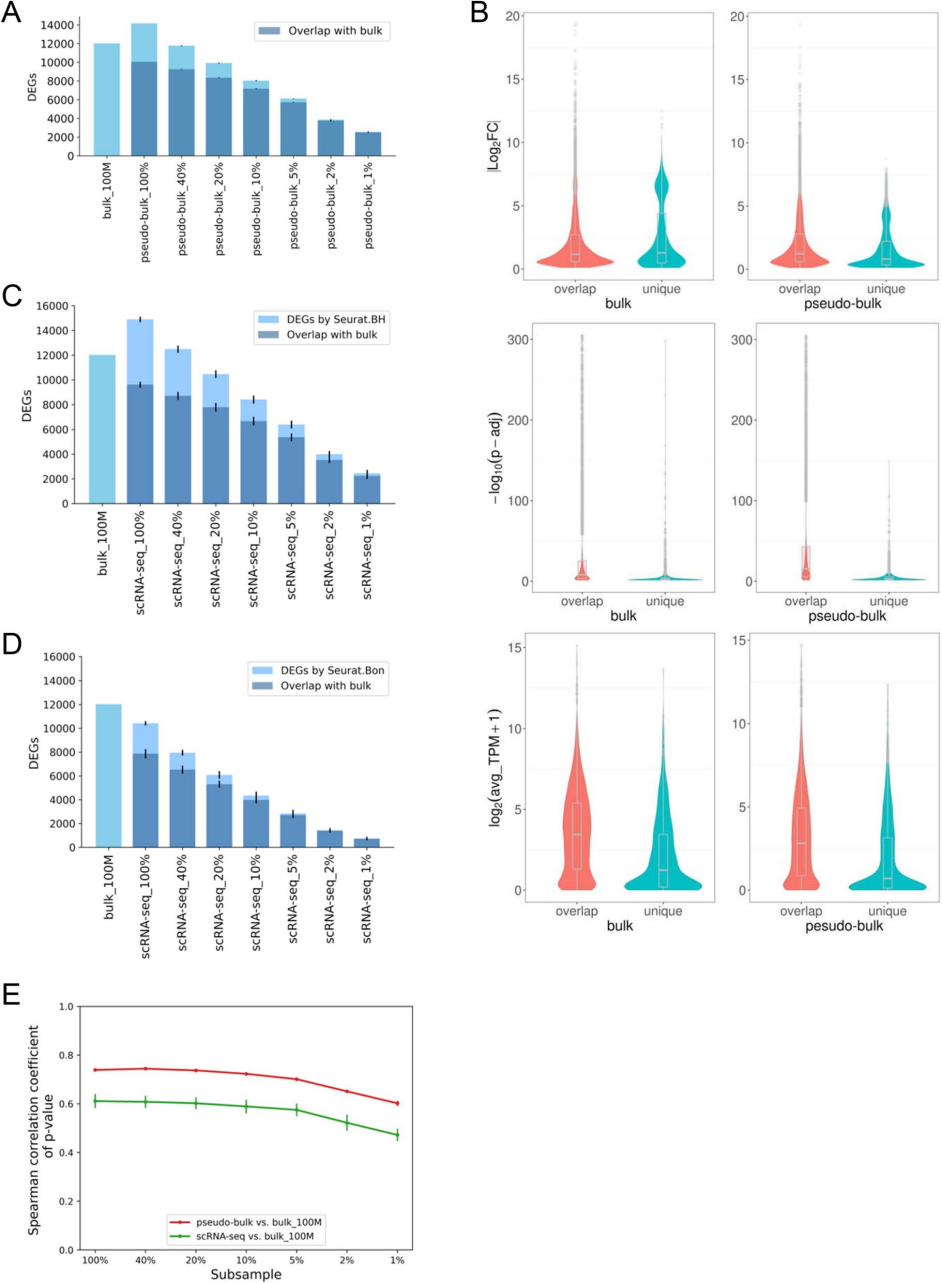
The number of cells substantially influences the identification of DEGs by scRNA-seq. **A** Overlap of DEGs identified by bulk and pseudo-bulk RNA-seq. **B** Characteristics of DEGs identified by both bulk and pseudo-bulk RNA-seq or by one method only. **C** Overlap of DEGs identified by bulk RNA-seq and by scRNA-seq analyzed with Seurat with BH adjustment. **D** Overlap of DEGs identified by bulk RNA-seq and by scRNA-seq analyzed with Seurat with Bonferroni adjustment. The bulk RNA-seq data were analyzed using BH adjustment. scRNA-seq analyzed using Bonferroni adjustment was plotted here for reference only as it was the default setting in Seurat. **E** Spearman correlation coefficients of *p* values of DEGs for bulk vs. pseudo-bulk RNA-seq data and bulk vs. scRNA-seq data. 100% to 1% corresponded to scRNA-seq data from approximately 5,000 to 50 cells, randomly sampled three time at each level below 100%. DEG, differentially expressed gene; Log_2_FC, log_2_ fold change; TPM, transcript per million; Q1 to Q4, first to fourth quartile. *N* = 3

DESeq2 analysis of the bulk RNA-seq data identified 12,027 DEGs between EC and VSMC. Analysis of the pseudo-bulk RNA-seq data using DESeq2 and direct analysis of single cell data using the default Wilcoxon Rank Sum test in Seurat identified a large majority (65% to 84%) of the DEGs identified by bulk RNA-seq and a few thousand DEGs not identified by bulk RNA-seq (Fig. 3A-D). Decreasing the number of cells included in the analysis of scRNA-seq substantially decreased the number of DEGs identified and the fraction of bulk RNA-seq-based DEGs that was recapitulated (Fig. 3A, C, D). The fraction of DEGs unique to scRNA-seq also decreased substantially. The Spearman correlation coefficients of *p* values of DEGs were 0.739 for bulk vs. pseudo-bulk RNA-seq data and 0.611 for bulk vs. scRNA-seq data. The correlations decreased modestly as the number of cells included in the pseudo-bulk and scRNA-seq data decreased (Fig. 3E).

**Fig. 4:**
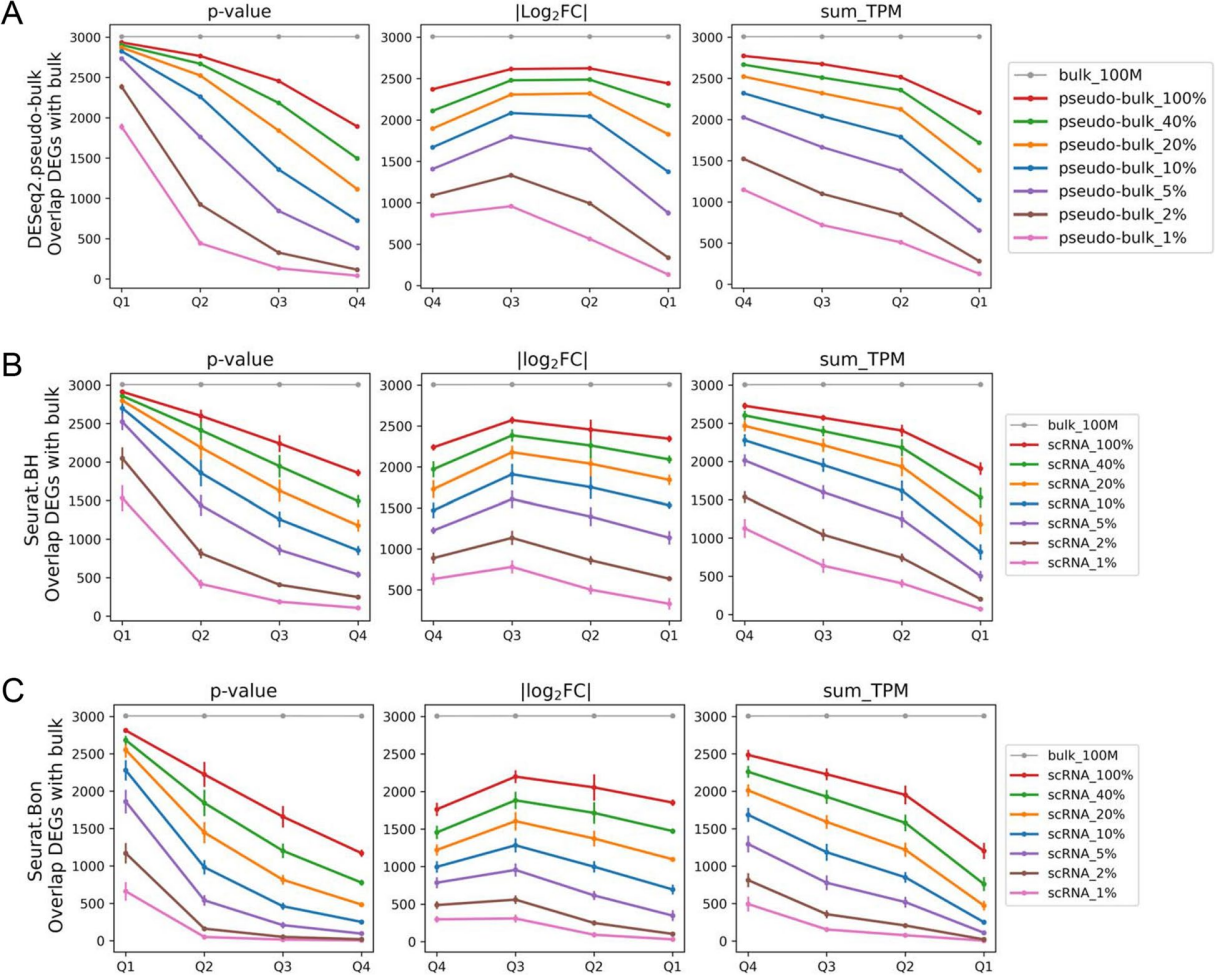
*P* value, fold change, and transcript abundance of DEGs identified by bulk RNA-seq compound the effect of cell number on the identification of the DEGs by scRNA-seq. Effects of p value (from small to large), absolute log-transformed fold change (from large to small), and transcript abundance (from high to low) on the overlap of DEGs identified by bulk and pseudo-bulk RNA-seq (**A**) or scRNA-seq analyzed with Seurat with BH adjustment (**B**) or Bonferroni adjustment (**C**). 100% to 1% corresponded to scRNA-seq data from approximately 5,000 to 50 cells, randomly sampled three time at each level below 100%. DEG, differentially expressed gene; Log_2_FC, log_2_ fold change; TPM, transcript per million; Q1 to Q4, first to fourth quartile. *N* = 3

The *p* value, fold change, and abundance of DEGs identified by bulk RNA-seq also affected the recapitulation of DEGs by scRNA-seq, and these effects compounded the effect of cell number (Fig. 4). The majority (> 50%) of DEGs in the quartile with smallest *p* values (unadjusted *p* < 2.8 × 10^–24^) was recapitulated by scRNA-seq based on 50 cells and analyzed as either pseudo-bulk or direct analysis with the BH adjustment (Fig. 4). The majority (> 50%) of DEGs in the quartile with highest transcript abundance (> 221 transcripts per million or TPM) was recapitulated by scRNA-seq based on 100 cells. The percent of DEGs recapitulated by scRNA-seq based on 100 cells dropped to below 10% for DEGs in the quartile with largest *p* values (unadjusted *p* > 7.0 × 10^–4^) and lowest transcript abundance (< 5.92 TPM).

**Fig. 5:**
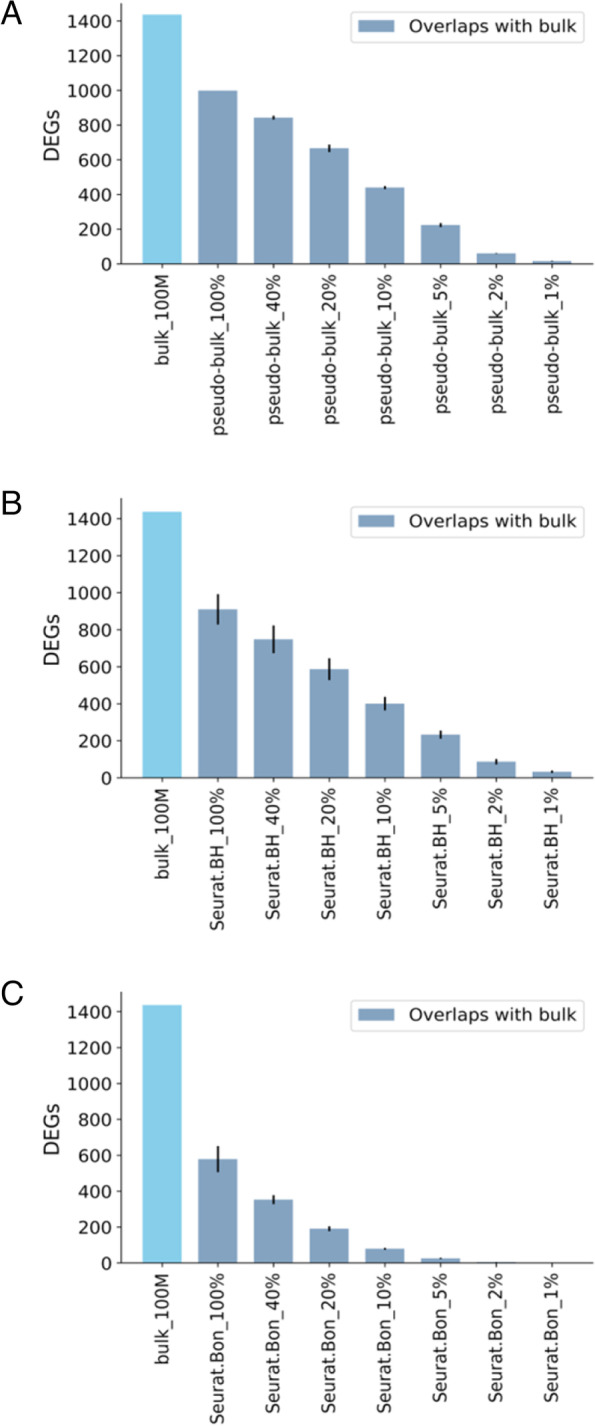
Identification of DEGs with modest changes by scRNA-seq. DEGs with modest changes referred to the 1,437 DEGs identified from the bulk RNA-seq that had unadjusted p between 3.1 × 10^–5^ and 0.025 (BH adjusted *p* values between 0.0001 and 0.05) and absolute log_2_ fold changes between 0.5 and 2 (i.e., 1.4 to 4 fold). **A** Recapitulation of modest DEGs by pseudo-bulk RNA-seq. **B** Recapitulation of modest DEGs by scRNA-seq analyzed with Seurat with BH adjustment. **C** Recapitulation of modest DEGs by scRNA-seq analyzed with Seurat with Bonferroni adjustment. The bulk RNA-seq data were analyzed using BH adjustment. scRNA-seq analyzed using Bonferroni adjustment was plotted here for reference only as it was the default setting in Seurat. 100% to 1% corresponded to scRNA-seq data from approximately 5,000 to 50 cells, randomly sampled three time at each level below 100%. DEG, differentially expressed gene. *N* = 3

Studies of a cell type under different experimental conditions often find DEGs with more modest fold changes and *p* values than studies comparing different cell types. We examined the 1,437 DEGs identified from the bulk RNA-seq with unadjusted p between 3.1 × 10^–5^ and 0.025 (adjusted *p* values between 0.0001 and 0.05) and absolute log_2_ fold changes between 0.5 and 2 (i.e., 1.4 to fourfold). Analysis of pseudo-bulk data based on approximately 5,000 cells identified 70% of these DEGs. The percentage remained above 50% (59%) with pseudo-bulk data from 2,000 cells but decreased to less than 10% with 100 cells (Fig. 5A). Results from direct analysis of the scRNA-seq data followed a similar trend (Fig. 5B, C).

**Fig. 6:**
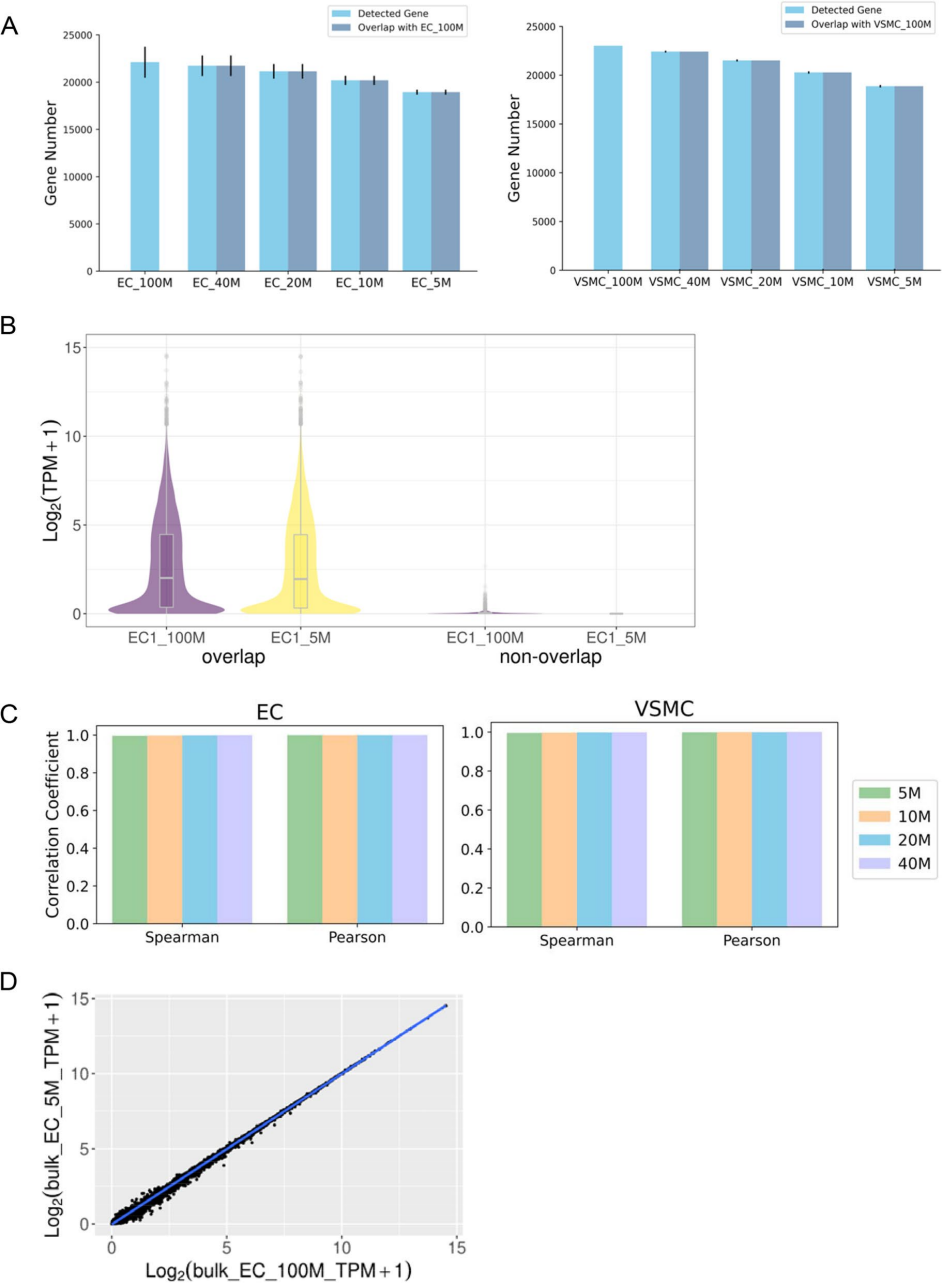
Transcriptome profiles from bulk RNA-seq with lower amounts of sequencing data were highly correlated with the original bulk-RNA-seq data. **A** Number of genes detected by various read numbers, and the overlaps with 100 M read pairs. **B** log_2_(TPM + 1) of genes detected by both 100 M and 5 M read pairs or by one level of read pairs only. **C.** Correlations of transcript abundance between 100 M and various sequencing depths. **D.** An example of the correlation. EC, endothelial cells; VSMC, vascular smooth muscle cells; TPM, transcript per million. *N* = 3

**Fig. 7:**
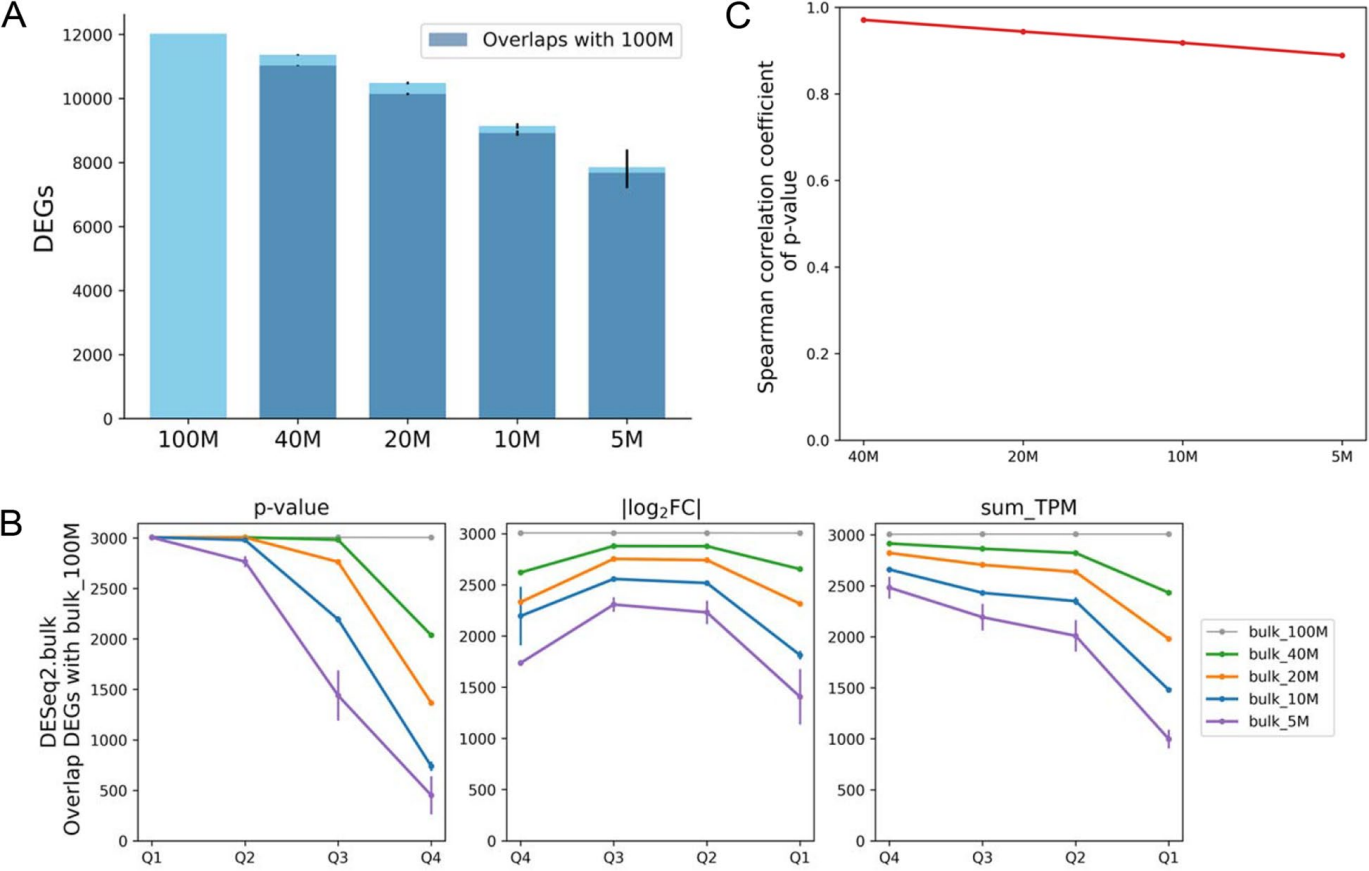
DEGs identified from bulk RNA-seq with lower amounts of sequencing data. Down-samples of 40, 20, 10, and 5 million read pairs were close to the number of read pairs from 5,000, 2,000, 1,000, and 500 cells, respectively, in scRNA-seq. **A** Overlap of DEGs identified from various amounts of data and overlaps with DEGs from 100 M read pairs. **B** Effect of p value, fold change, and abundance on the overlap of DEGs identified by lower amounts of data with DEGs from 100 M read pairs. **C** Spearman correlation coefficients of *p* values of DEGs for 100 M read pairs vs. the down-samples. DEG, differentially expressed gene; log_2_FC, log_2_ fold change; TPM, transcript per million; Q1 to Q4, first to fourth quartile. *N* = 3

We randomly down-sampled the bulk RNA-seq data to examine the effect of sequencing depth. (Supplemental Table S2). The overall transcript profile at each level of down sampling was highly similar with the original sample (Fig. 6), in contrast with the modest similarity between pseudo-bulk and bulk RNA-seq data (see Fig. 2). The numbers of DEGs recapitulated with lower amounts of sequencing data were 7% to 10% greater than the numbers recapitulated by scRNA-seq data with a similar number of read pairs (Fig. 7A; compared with Fig. 3). The p value, fold change, and abundance of DEGs also affected the recapitulation of DEGs with lower amounts of sequencing data (Fig. 7B). The Spearman correlation coefficient of *p* values of DEGs was 0.971 for 100 M read pairs vs. 40 M, decreasing to 0.889 for 5 M (Fig. 7C). These correlations were substantially higher than the correlations between bulk and pseudo-bulk or scRNA-seq data shown in Fig. 3E.

## Discussion and conclusions

Findings of the current study provide a quantitative reference for designing studies that aim for identifying DEGs for specific cell clusters using scRNA-seq data and for interpreting results of such studies. If a study anticipates DEGs with modest differences, the study should aim for having 2,000 or more cells in a cluster in order to identify the majority of DEGs that would have been identified by a bulk RNA-seq analysis of thousands of physically purified cells. Such studies should be cautious in interpreting a lack of DEGs from clusters with fewer than 100 cells. On the other hand, clusters with as few as 50–100 cells may be sufficient for identifying the majority of DEGs that would have extremely small *p* values or transcript abundance greater than a few hundred TPM in a bulk RNA-seq analysis.

Our study was performed in two cell types derived from one iPSC line, and one should be cautious in extrapolating our findings directly to other cell types. However, our findings are likely to be relevant to a wide range of experimental scenarios as we tested various sizes of dataset and degrees of differential expression. scRNA-seq analysis remains several fold more expensive than bulk RNA-seq and requires additional expertise and effort. The quantitative reference provided by the current study should be an important consideration for scRNA-seq studies.

The effect of cell number in scRNA-seq on the recapitulation of DEGs appears largely, but not completely, explainable by the effect of total sequencing depth. In addition, the analysis of scRNA-seq data identifies new DEGs not identified by bulk RNA-seq. It is not clear whether these new DEGs are false positives from the scRNA-seq analysis or false negatives in the bulk RNA-seq analysis.

## Methods

### Differentiation of iPSC to EC and VSMC

The human iPSC line 039B used in this study was reprogrammed from urine cell from a 35-year-old female Caucasian using Sendai virus following the protocol described previously [8]. All procedures were approved by the Institutional Review Boards at the Medical College of Wisconsin with patient consent. iPSCs were differentiated into EC and VSMC following previously published protocols [9] with modifications. Briefly, 039B iPSCs were cultured on Matrigel coated dishes with mTeSR™ plus (STEMCELL Technologies) on 6-cm dishes and routinely passaged at a dilution of 1:6 to 1:10. For differentiation, iPSCs were dissociated using Accutase (STEMCELL Technologies) and plated on Matrigel coated 6-well plates at a density of 47,000 cells/cm^2^ in mTeSR™ plus with 10 µM Rock inhibitor Y-27632 (STEMCELL Technologies). After 24 h, cells were treated with N2B27 medium (a 1:1 mixture of DMEM:F12 with Glutamax and Neurobasal media supplemented with N2 supplement and B27 supplement minus vitamin A; all Life Technologies) plus 8 µM CHIR99021 (Selleck Chemicals) and 25 ng/ml BMP4 (PeproTech) for 3 days to generate mesoderm cells. ECs were further induced with StemPro-34 SFM medium (STEMCELL Technologies) supplemented with 200 ng/ml VEGF (PeproTech) and 2 µM forskolin (Abcam) for 2 days and purified with CD144 magnetic beads (Miltenyi Biotec). CD144-positive cells were cultured in StemPro-34 SFM medium supplemented with 50 ng/ml VEGF for 5 days before harvest. For VSMC induction, mesoderm cells were treated with N2B27 medium supplemented with 10 ng/ml PDGF-BB (PeproTech) and 2 ng/ml Activin A (PeproTech) for 2 days. Contractile VSMCs were then induced with N2B27 supplemented with 2 ng/ml Activin A and 2 µg/ml Heparin (STEMCELL Technologies) for 5 days. VSMCs were enriched by removing CD144 + cells using CD144 magnetic beads.

### scRNA-seq library preparation and sequencing

scRNA-seq library preparation was performed using Chromium Next GEM Single Cell 3ʹ Reagent Kits v3.1 (Dual Index) (10 × Genomics). The libraries were subjected to 150 bp paired-end sequencing using NovaSeq 6000 with the v1.5 S4 reagent kit and Flowcell (Novogene).

### scRNA-seq data processing and sampling

Single cell feature counts were generated by cellranger count (Cell Ranger v6.0.0, 10 × Genomics) with sequencing reads in FASTQ files and the human reference GRCh38 dataset. To remove the ambient RNA from count matrices, we used remove-background from CellBender v0.2.1 with FPR = 0.01. The analysis was performed on an HPC GPU cluster in the Research Computing Center at the Medical College of Wisconsin. Cells with fewer than 200 or more than 5,000 detected genes were filtered out [10]. Seurat R package v4.1.1 was used for downstream data processing including clustering [6]. Random sampling of a desired percentage of cells was repeated three times from each scRNA-seq library to examine the effect of cell number.

### Bulk RNA-seq library preparation and sequencing

Total RNA was extracted from iPSC-derived ECs or VSMCs with TRIzol reagent (Thermo Fisher). Libraries for poly(A)-dependent RNA-seq, which will be called RNA-seq for convenience in this article, were prepared with NEBNext Ultra II RNA Library Prep kit following its Section I protocol (Novogene). The libraries were subjected to 150 bp paired-end sequencing using NovaSeq 6000 with the v1.5 S4 reagent kit and Flowcell (Novogene).

### Bulk RNA-seq data processing and sampling

We used Trim Galore v0.6.6 to trim the adapter and low-quality raw reads. Random sampling of a desired number of sequencing reads was done using the Seqtk tool (v1.3). Reads were then aligned to the human reference genome GRCh38-2020-A with STAR tools (v2.7.9a) [11]. Reads per gene were counted by htseq-count of htseq v0.13.5 [12].

### Statistical analysis

We took read count matrix as input for all the data analysis. For each sample, we computed pseudo-bulk data from scRNA-seq count matrix with the sum of counts mapped to each gene. Transcript per million (TPM) value was used to quantify expression levels of genes as defined in GENCODE GRCh38.p13 for bulk and pseudo-bulk data. DESeq function from DESeq2 v1.34.0 was used to identify DEGs between EC and VSMC for bulk RNA-seq data and pseudo-bulk data [13]. Benjamini-Hochberg (BH) adjusted *p*-value equal to or lower than 0.05 was considered significant. Default settings were used for all other parameters.

For direct analysis of scRNA-seq data, we used Seurat v4.1.1 to identify differentially expressed features. EC and VSMC were merged into one Seurat object as two identities. We normalized Seurat objects with “LogNormalize” method and set scale factor to 10,000. Since we wanted to include all genes and cells for comparison with bulk RNA-seq, we set logfc.threshold = 0 and min.pc = 0. *P*-values were calculated by the FindMarkers function using the default Wilcoxon Rank Sum test. DEGs between EC and VSMC were identified by Bonferroni and, separately, Benjamini- Hochberg (BH) adjusted p value equal to or lower than 0.05. scRNA-seq data from randomly sampled cells were processed in the same way.

All the data analysis was performed in R v4.1.1. Violin plots were generated using ggplot2 v3.3.6 [14]. Venn plots and bar plots were created by Matplotlib library [15] in python v3.8.13.

## Supplementary Information

**Additional file 1: Supplemental Table S1.** Characteristics of the bulk RNA-seq data. **Supplemental Table S2.** Characteristics of the scRNA-seq data. **Supplemental Figure S1.** The vast majority of cells in each scRNA-seq library formed one cluster in an UMAP plot and expressed a marker gene for the cell type.

## Data Availability

The datasets generated and/or analyzed during the current study have been deposited in NCBI's Gene Expression Omnibus and are accessible through GEO Series accession number GSE226163.
