# Supplementary material for: Robustness of single-cell RNA-seq for identifying differentially expressed genes
Source: BMC Genomics. 2023 Jul 3;24:371. doi: 10.1186/s12864-023-09487-y (PMC10316566; doi:10.1186/s12864-023-09487-y)
Supplement: Supplementary file 1 — Additional file 1: Supplemental Table S1. Characteristics of the bulk RNA-seq data. Supplemental Table S2. Characteristics of the scRNA-seq data. Supplemental Figure S1. The vast majority of cells in each scRNA-seq library formed one cluster in an UMAP plot and expressed a marker gene for the cell type. [file 12864_2023_9487_MOESM1_ESM.pdf]

Supplemental Materials

Supplemental Table S1 and S2

Supplemental Figure S1

Supplemental Table S1. Characteristics of the bulk RNA-seq data.

Sample	Read counts	Raw data (GB)	Effective (%)	Error (%)	Q20 (%)	Q30 (%)	GC (%)	Number of input reads (pair)	Uniquely mapped reads number (pair)	Uniquely mapped reads (%)
EC1	205,478,712	30.8	97.44	0.03	97.46	93.36	52.22	102,978,073	93,347,489	90.65
EC2	176,370,990	26.5	97.49	0.03	97.40	93.39	53.54	101,225,035	94,259,773	93.12
EC3	243,485,930	36.5	97.74	0.03	97.66	93.82	51.99	110,261,305	92,020,788	83.46
VSMC1	202,531,772	30.4	98.50	0.03	97.64	93.74	51.56	88,149,116	71,988,614	81.67
VSMC2	206,041,232	30.9	98.01	0.03	97.54	93.52	51.54	102,693,341	90,714,129	88.33
VSMC3	220,614,750	33.1	97.77	0.03	97.28	93.14	53.21	121,696,793	109,948,232	90.35

Supplemental Table S2. Characteristics of the scRNA-seq data.

Data after CellBender				
Sample	# of cells	# of read pairs	Mean # of genes per cell	Mean # of read pairs per cell
EC1	4,174	71,243,125	4,700	17,068
EC2	6,016	78,133,493	4,113	12,988
EC3	7,653	89,154,306	3,831	11,650
VSMC1	5,994	69,424,851	3,216	11,582
VSMC2	6,287	86,500,831	3,475	13,759
VSMC3	6,362	79,947,712	3,286	12,566
Data after CellBender and including cells with more than 200 and less than 5,000 genes				
Sample	# of cells	# of read pairs	Mean # of genes per cell	Mean # of read pairs per cell
EC1	2,372	26,182,335	3,807	11,038
EC2	4,465	44,324,608	3,598	9,927
EC3	6,146	55,498,556	3,394	9,030
VSMC1	5,706	61,773,818	3,107	10,826
VSMC2	5,798	71,849,634	3,294	12,392
VSMC3	5,995	69,271,877	3,149	11,555

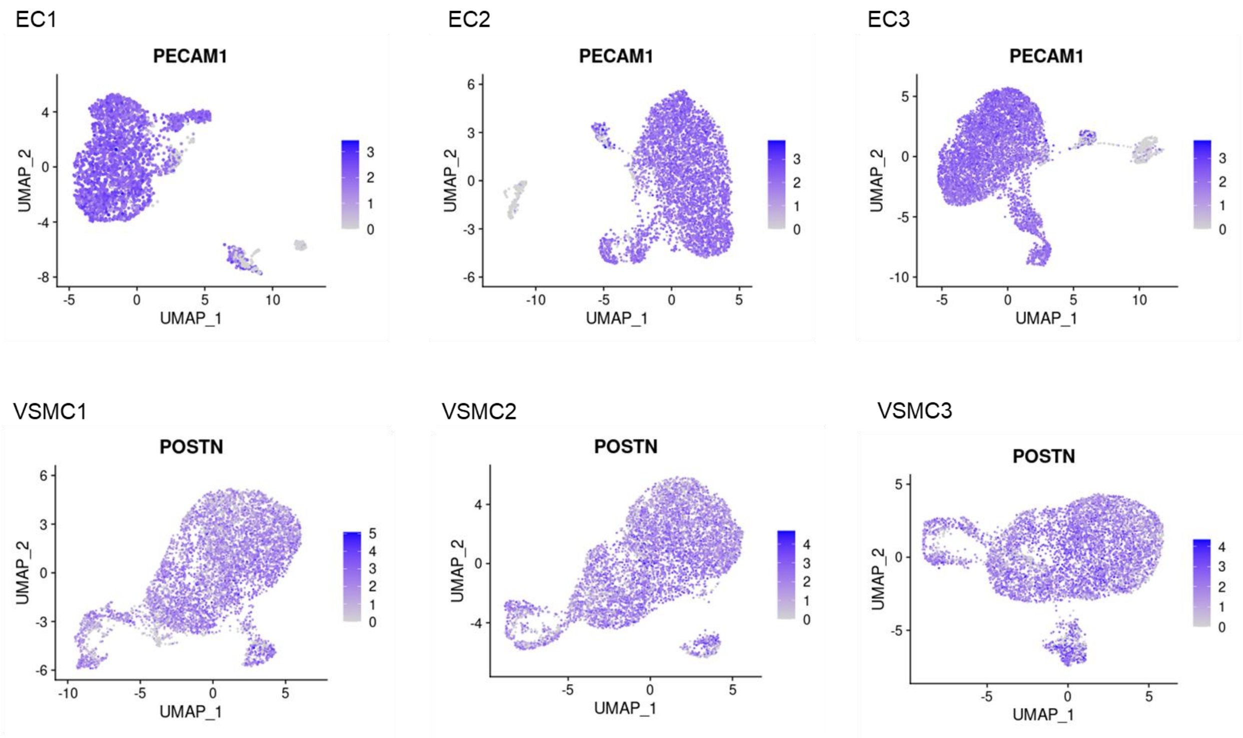

Supplemental Figure S1. The vast majority of cells in each scRNA-seq library formed one cluster in an UMAP plot and expressed a marker gene for the cell type. The shared nearest neighbor (SNN) modularity optimization-based clustering algorithm in Seurat was used. UMAP, Uniform Manifold Approximation and Projection; EC, iPSC-derived endothelial cells; VSMC, iPSC-derived vascular smooth muscle cells.
